# Diagnosis of Chagasic Encephalitis by Sequencing of 28S rRNA Gene

**DOI:** 10.3201/eid2507.180285

**Published:** 2019-07

**Authors:** Ashrit Multani, Aabed Meer, Darvin S. Smith, Malika N. Kheraj, Edward D. Plowey, Brian G. Blackburn

**Affiliations:** Stanford University School of Medicine, Stanford, California, USA (A. Multani, A. Meer, E.D. Plowey, B.G. Blackburn);; Kaiser Permanente Northern California, Redwood City, California, USA (D.S. Smith, M.N. Kheraj)

**Keywords:** chagasic encephalitis, Chagas disease, Trypanosoma cruzi, parasites, protozoa, meningitis/encephalitis, 28S rRNA gene, rRNA sequencing, HIV/AIDS and other retroviruses, immunocompromised

## Abstract

We report a case of chagasic encephalitis diagnosed by 28S rRNA sequencing. The diagnosis of chagasic encephalitis is challenging, given the broad differential diagnosis for central nervous system lesions in immunocompromised patients and low sensitivity of traditional diagnostics. Sequencing should be part of the diagnostic armamentarium for potential chagasic encephalitis.

Chagasic encephalitis is a rare disease in the United States. We report a case of chagasic encephalitis in an HIV-infected man. This case was diagnosed by sequencing of the parasite 28S rRNA gene.

## The Study

The patient was a 31-year-old HIV-infected man who had fevers, headaches, and ataxia for 3 weeks. He had lived in El Salvador until moving to the United States 6 years earlier. His neurologic symptoms persisted, and he was hospitalized after cranial computed tomography (CT) showed a 6-cm, heterogeneous, centrally necrotic mass in the corpus callosum. At admission, he was afebrile, oriented only to self, and had slow movements.

**Figure 1:**
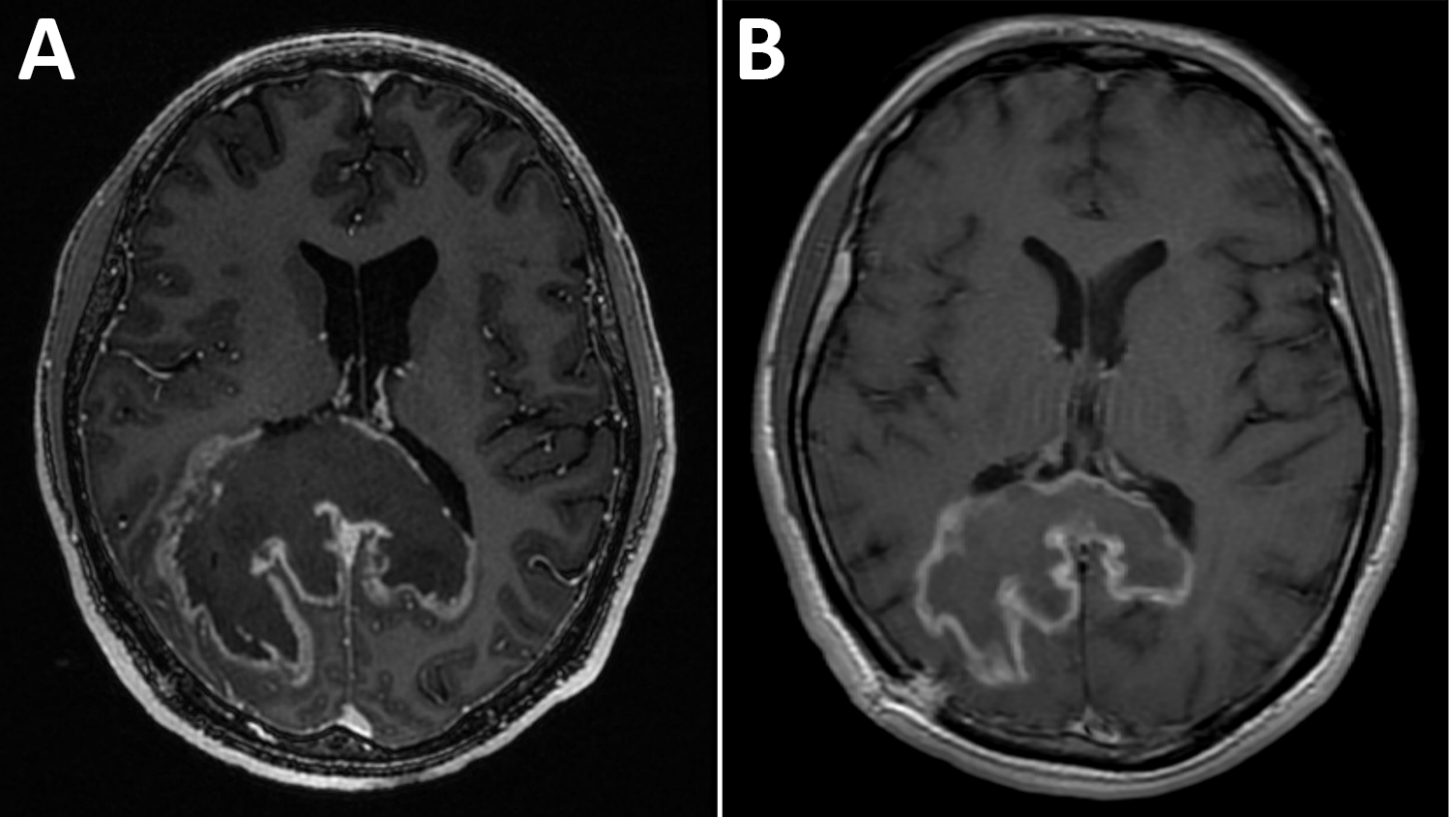
Images obtained during diagnosis of chagasic encephalitis in 31-year-old man in the United States. A) Contrast-enhanced T1-weighted magnetic resonance imaging of the brain showing a cerebral tumor-like chagoma in the axial plane. B) Follow-up contrast-enhanced T1-weighted magnetic resonance imaging obtained ≈8 weeks later showing improvement of the chagoma.

Testing showed a leukocyte count of 3,500 cells/μL, hemoglobin level of 12.4 g/dL, CD4 cell count of 60 cells/μL, HIV viral load of 409,302 copies/mL, and a positive result for serum *Toxoplasma gondii* IgG. Chest radiograph results were unremarkable. Magnetic resonance imaging (MRI) of the brain (Figure 1, panel A) showed an 8.1 × 7.3 cm heterogeneous mass centered within the corpus callosum and parietal–occipital subcortical white matter. Diffusion restriction was identified mostly within peripheral portions of the lesion. Administration of gadolinium showed heterogeneous peripheral enhancement and central necrotic change. Additional foci of abnormal fluid-attenuated inversion recovery signal and enhancement were noted within posterior fossa and supratentorial parenchyma. On the basis of these findings, radiologically favored diagnoses included lymphoma, glioblastoma, or tumefactive multiple sclerosis. Infection was believed less likely, given the absence of prominent central diffusion restriction.

Urgent MRI-directed stereotactic biopsy of the brain was performed. Cytologic smear preparations showed an intraoperative pathological impression of toxoplasmosis on the basis of identification of protozoal organisms. The patient was given trimethoprim/sulfamethoxazole for possible toxoplasmic encephalitis while we awaited procurement of pyrimethamine/sulfadiazine.

**Figure 2:**
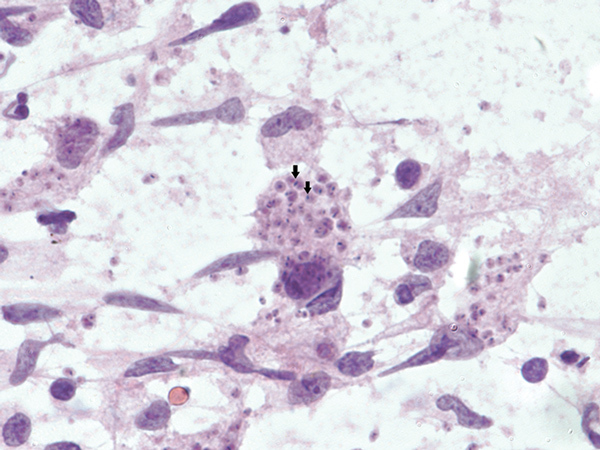
Tissues obtained during diagnosis of chagasic encephalitis in 31-year-old man in the United States. Touch preparation of brain tissue showing necrotizing encephalitis and abundant *Trypanosoma cruzi* amastigotes with prominent kinetoplasts (arrows) in astrocytes and macrophages (hematoxylin and eosin stain, original magnification ×600).

**Figure 3:**
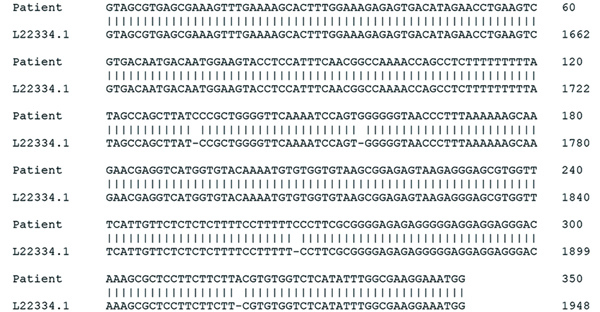
Sequences obtained during diagnosis of chagasic encephalitis in 31-year-old man in the United States. Alignment of amplicon sequence isolated from the patient was compared with a reference *Trypanosoma cruzi* 5.8S rRNA internal transcribed spacer sequence (GenBank accession no. L22334.1). Percentage identity was 98.9% (346/350 bases). Hyphens indicate gaps in the genome.

Subsequent review of permanent pathologic sections showed necrotizing encephalitis and abundant amastigotes with prominent kinetoplasts in astrocytes and macrophages (Figure 2). Immunostaining for *Toxoplasma* spp. was negative. Sequencing of the internal transcribed spacer 2 and D2 regions of the 28S rRNA gene in paraffin-embedded tissue identified the organism as *Trypanosoma cruzi* (Figure 3) (*1*,*2*). A *T. cruzi* IgG test result was subsequently positive; results of peripheral blood smear examination were negative for circulating trypomastigotes.

Trimethoprim/sulfamethoxazole was decreased to prophylactic dosing, and benznidazole (2.5 mg/kg 2×/d) was given after receipt of this drug from the Centers for Disease Control and Prevention (Atlanta, GA, USA) Drug Service 6 days after admission. Treatment with lamivudine, zidovudine, and nevirapine was begun 1 week later.

The patient’s course was complicated by leukopenia requiring benznidazole treatment interruption and replacement of zidovudine with abacavir. He completed 60 days of benznidazole therapy over a 3-month period but did not receive secondary prophylaxis for *T. cruzi*. Three months after initial presentation, his HIV viral load was suppressed, his CD4 count had increased sustainably to >200 cells/μL, he was symptomatically and radiologically (Figure 1, panel B) improved, and he returned to work.

## Conclusions

*T. cruzi*, the causative agent of Chagas disease, is endemic to much of Latin America (*3*). Most patients infected with *T. cruzi* remain asymptomatic for years to decades, after which cardiac or gastrointestinal complications develop in some patients. In immunocompromised patients, when Chagas disease manifests clinically, it occurs most commonly because of reactivation of latent *T. cruzi* infection (*4*–*7*). Although rare in other cohorts, central nervous system (CNS) involvement is the most common manifestation of Chagas disease in patients with AIDS; chagasic encephalitis is also found in patients with other forms of immunosuppression (*4*–*8*). Chagasic encephalitis usually manifests as an abscess but can also manifest as meningoencephalitis; signs and symptoms include headache, focal neurologic deficits, fever, meningismus, seizures, and altered mentation (*4*–*8*).

Differential diagnosis of a CNS mass lesion in a patient with AIDS is broad. Brain MRI usually shows a single, large, tumor-like ring-enhancing lesion in patients with chagasic encephalitis (*4*–*8*). Imaging studies cannot distinguish chagasic encephalitis in this cohort from other common CNS conditions, including toxoplasmosis, lymphoma, progressive multifocal leukoencephalopathy, tuberculoma, cryptococcoma, nocardiosis, and pyogenic abscess (*4*–*9*). For this patient, imaging findings favored malignancy (lack of central diffusion restriction was particularly atypical of an infection). The diagnosis of toxoplasmosis was considered before chagasic encephalitis, given the relative frequency of these diagnoses in the United States, *T. gondii* seropositivity, and the preliminary pathological interpretation. These factors highlight the difficulty of accurate diagnosis of chagasic encephalitis and that more common confounders are likely to be considered before chagasic encephalitis, a rare diagnosis in the United States.

Because most cases of chagasic encephalitis in AIDS patients occur because of reactivation of chronic infection, patients are usually positive for *T. cruzi* IgG at the time of diagnosis; a negative result argues against this possibility (akin to the role of serology in assessing the likelihood of toxoplasmic encephalitis in AIDS patients). Conversely, although a negative result for *T. cruzi* IgG carries a high negative predictive value for chagasic encephalitis, the predictive value of a positive result for IgG is lower because patients might be seropositive from past infection unrelated to their current CNS process. *T. cruzi* parasitemia can also be detected (by microscopic examination of peripheral blood) in some patients with AIDS who have chagasic encephalitis, obviating the need for a brain biopsy, although the predictive value is not high enough to exclude chagasic encephalitis in patients with a negative result.

Conventional PCR performed on blood is not useful for diagnosing *T. cruzi* reactivation because the result can be positive for patients with chronic *T. cruzi* infection without reactivation (although quantitative PCRs performed on serial blood specimens that show increasing parasite copy numbers over time can indicate reactivation) (*10*,*11*). Given these issues, confirmation of chagasic encephalitis often requires direct microscopic visualization of the organism (*5*–*8*). Unfortunately, *T. cruzi* is difficult to identify microscopically because polymorphism is common, resulting in confusion with other organisms and leading to the need for better confirmatory tests (*12*).

One report described use of molecular testing of cerebrospinal fluid or brain tissue to establish the diagnosis of chagasic encephalitis (*13*). Although some laboratories use real-time PCRs for molecular detection of *T. cruzi* DNA, only this pathogen can be detected in this way, and a negative PCR result for *T. cruzi* in a tissue sample does not reliably exclude the diagnosis because of low sensitivity (*14*). Because multiple infectious diseases in this scenario can be indistinguishable clinically and radiologically, a high index of suspicion and a battery of organism-specific tests are required for comprehensive evaluation. In contrast, because the D2 primers used in sequencing react with multiple protozoa and fungi, it can detect not only *T. cruzi* but also other pathogens that share the D2 subunit, such as *T. gondii*, *Cryptococcus* spp., and *Histoplasma* spp. (and *Leishmania* spp. in other clinical settings) (*2*). The ability to identify one of many potential pathogens with a single test is advantageous for timely institution of appropriate treatment and patient outcome.

As a result of increasing urbanization and globalization, migration continues from areas with high prevalence of *T. cruzi* to nonendemic areas (*3*). Also, increasing use of immunomodulatory therapies, cancer chemotherapeutics, and solid organ and hematopoietic cell transplantation places increasing numbers of patients chronically infected with *T. cruzi* at risk for reactivation. Diagnosing CNS processes in these patients is challenging because of nonspecific clinical and radiologic findings and a broad differential diagnosis, in addition to inherent limitations of traditional diagnostic tests. Chagasic encephalitis is a life-threatening condition that should be included in the differential diagnosis for immunocompromised patients from disease-endemic areas who have cerebral mass lesions or meningoencephalitis. Newer diagnostic methods, such as rRNA gene sequencing, can enable rapid diagnosis and should be considered as part of the diagnostic armamentarium.
